# Dynamics of soil ingestion by growing bulls during grazing on a high sward height in the French West Indies

**DOI:** 10.1038/s41598-020-74317-0

**Published:** 2020-10-14

**Authors:** Claire Collas, Maurice Mahieu, Pierre-Marie Badot, Nadia Crini, Guido Rychen, Cyril Feidt, Stefan Jurjanz

**Affiliations:** 1 Unité de Recherche Animal et Fonctionnalités des Produits Animaux, Université de Lorraine, INRAE, URAFPA, 54000 Nancy, France; 2 Unité de Recherches Zootechniques, Institut National de Recherche pour l’Agriculture, l’Alimentation et l’Environnement (INRAE) Centre Antilles-Guyane, UR 0143, 97170 Petit-Bourg (Guadeloupe), France; 3 Laboratoire Chrono-Environnement, Université de Bourgogne Franche-Comté, CNRS, UMR 6249, 25030 Besançon Cedex, France

**Keywords:** Animal behaviour, Ecosystem services, Ecology, Zoology, Environmental sciences

## Abstract

Free-range livestock are exposed to environmental contaminants by ingesting contaminated matrices mainly soil. Several works evaluated precisely the soil ingestion and its variation factors in ruminants. Contrary to temperate grazing systems, tropical ones were poorly documented whereas weather or traditional grazing practices may change models established in temperate systems. The study was performed in the French West Indies, which are concerned by a widespread environmental chlordecone contamination. The work evaluated daily soil and grass ingestions by tethered growing bulls grazing on a very high sward close to 50 cm for 11 days without being moved. This grazing management is representative to local practices by small farmers or not professional holders and allows completing the results previously obtained. Daily soil ingestion did not significantly increase across time and was on average 26.9 g dry matter/100 kg body weight (i.e. 1.4% of the total mass ingested). Marked individual variations indicated that exposure risk assessments would require experimental designs based on a sufficient number of individuals. This study was also the first to investigate the changes in sward soiling with respect to the distance from the stake and reported lower soil loading on grass in the peripheral than central and intermediate areas.

## Introduction

Soil ingestion represents a major risk of exposure to environmental pollutants for free range animals without any supply of bioavailable nutrients^1,2^. In the French West Indies, 1/5th of the useful agricultural area of Guadeloupe and 2/5th of that of Martinique^3^ are contaminated by chlordecone, a very persistent organochlorine insecticide against banana weevil forbidden since 1993. This environmental contamination threatens the food safety of livestock products and the sustainability of farming activities^4^. Estimating soil ingestion by livestock in different grazing conditions will allow to establish grazing management recommendations to limit animal exposure to contaminants in soil, especially chlordecone.

The soil ingestion by grazing tethered cattle has been studied in Caribbean systems and ranged from 50 to 100 g dry matter (DM)/100 kg body weight (BW)/day^5,6^ with an increase when daily herbage allowance decreases. For similar pregrazing sward heights, it was observed that the postgrazing sward height was lower for animals receiving a restricted herbage allowance than for animals receiving a higher herbage allowance (3.3 vs 5.2 cm^5^; 6.3 vs 8.2 cm^6^). These results suggest that restricted animals would graze closer to the ground and could be subjected to a higher soil ingestion, so sward height can be a grazing management tool to limit soil ingestion.

**Figure 1:**
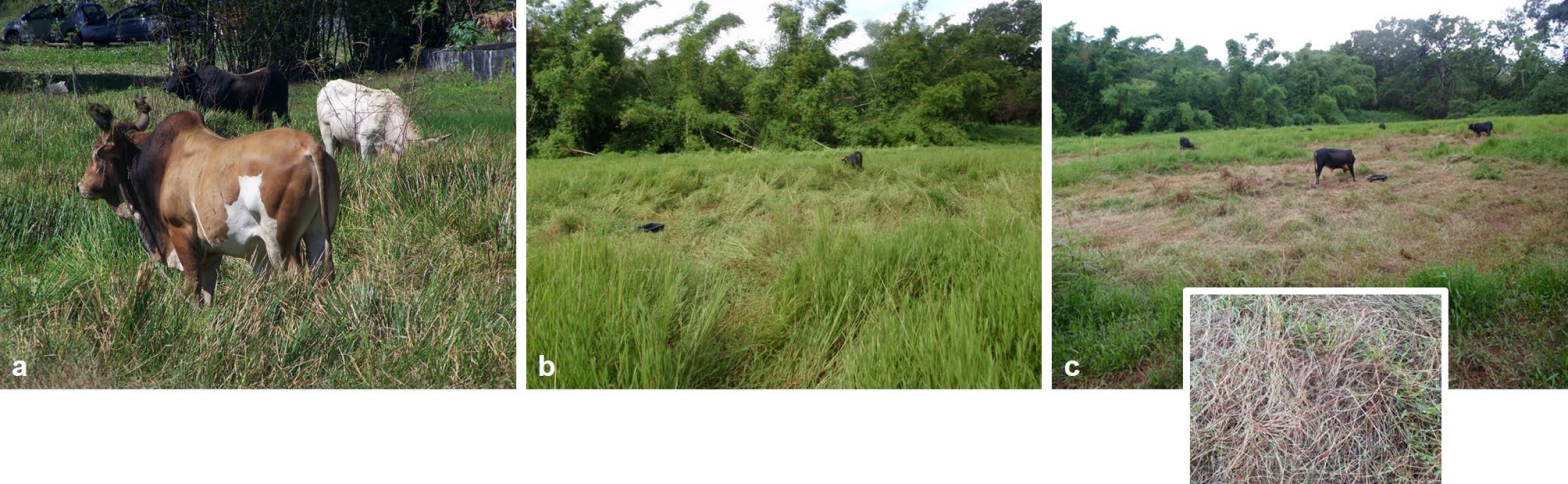
(**a**) Tether-grazing cattle in Guadeloupe with long chain and high sward in non-experimental conditions. (**b**) Experimental design reproducing such grazing conditions to evaluate daily evolution of soil ingestion, the 2nd day in the experimental plot (D3), (**c**) the 11th day in the experimental plot (D12).

These previous studies were performed using tethered animals daily moved to a new grazing spot on modest sward heights (i.e. 8–11 cm). Tethered-grazing is a common practice in Caribbean especially for not professional private holders^7^. However, it can be conducted differently when available biomass is very high, with tall forage species often reaching a height over 0.5 m above the ground, as in ancient banana plantations potentially polluted by chlordecone. In such areas, many cattle holders move them only once or twice a week, tying them with chains long enough to give access to an area sufficient to match their requirements (Fig. 1). Considering obvious differences between these grazing conditions and those previously studied, results from daily moved tether posts cannot be extrapolated on these grazing systems.

Collas et al.^6^ tested different daily herbage allowances when animals grazed on a water-saturated or a dried ground and reported a higher soil ingestion when soil surface was wet. They quantified soil loading on grass offered to animals and observed that sward soiling, due to animal trampling or movement of the chain on the ground, was amplified in the case of water-saturated soil or herbage allowance reduction. Therefore, the present work aimed to study for the first time the effect of such long-time placement of the tethering post and a very high pregrazing sward height on the kinetics of sward soiling and by consequence soil ingestion. Furthermore, this study targeted also to understand better the influence of grazing on spatial and temporal variations of soil loading on grass.

## Results and discussion

The aim of this study was to evaluate the kinetic of daily soil ingestion by growing bulls at tether-grazing when they received a very high sward and a large grazing area in which they stay during 11 days (no stake moving).

### Use of herbage resource

**Figure 2:**
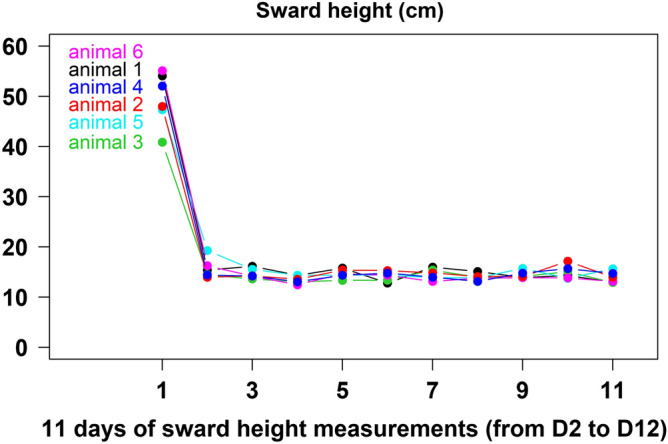
Evolution of the sward heights measured daily from D2 to D12 in each grazing circle of each animal. Each color represents the average daily sward heights for each grazing circle of each animal.

The average sward height was 17.6 ± 0.3 cm (mean ± s.e.m.) along the 11 days of measurements from D2 to D12. Nevertheless, pregrazing sward height (at D2) was quite high with 49.2 ± 0.9 cm and significantly higher from those on D3 to D12 (14.4 ± 0.1 cm; *P* < 0.001) with a 68% decrease along the first 24 h (15.6 ± 0.6 cm at D3; Fig. 2). On the last day (D12) on the experimental plot, the sward height was 13.9 ± 0.5 cm corresponding to a reduction of about 72% in 10 days (Fig. 2). This postgrazing sward height near to 14 cm seemed to be relatively high compared to the range of 3.3–8.2 cm reported in previous works^5,6^ but the important differences in postgrazing vegetation structure (long lying stems vs. short erected ones) limiting the comparison of postgrazing sward heights with these studies. Indeed, animals grazed on the experimental plot until resource depletion and at D12 the remaining vegetation cover was only constituted of lying fibrous stems forming an ‘insulating carpet’ above the ground (Fig. 1c). Sward heights decreased rapidly, mainly due to trampling by animals and the movements of the chain, and reflected more a rapid change in the structure of the vegetation (grass lying on the ground) than in the forage supply. Grass allowance decreased across time, but more progressively than sward heights, and the animals were moved to a new plot on D13 when they had ingested all the leafy parts (daily observation of the plot) to avoid any limitation to meet their nutritional requirements.

**Figure 3:**
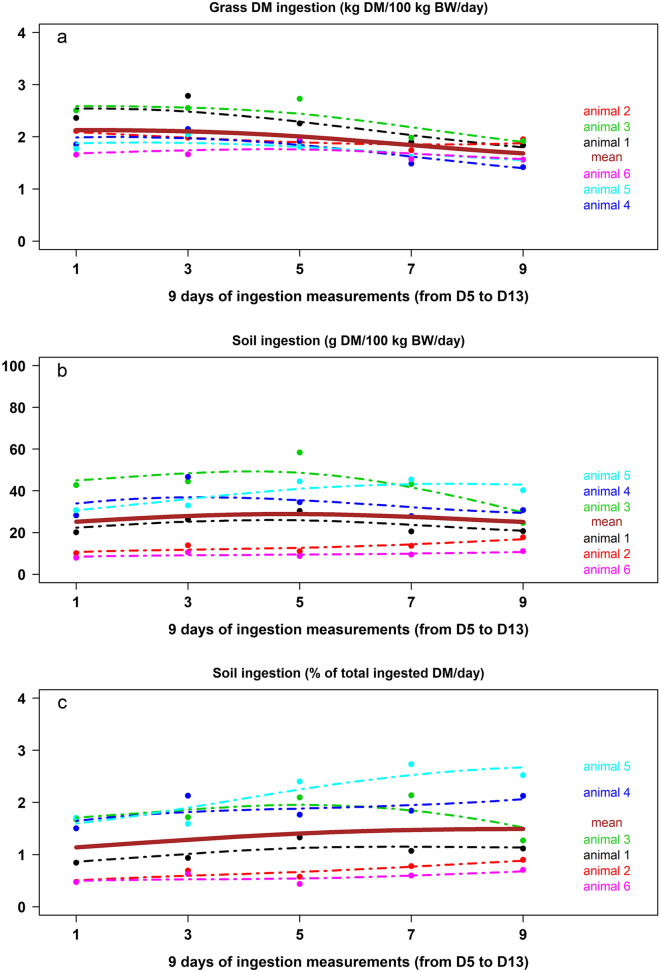
Evolution of individual (**a**) daily grass dry matter (DM) ingestion, (**b**) daily soil ingestion (g DM/100 kg BW), (**c**) daily soil ingestion (% of total ingested DM) during the 9 days of measurements (from D5 to D13) (*BW* body weight).

Daily grass ingestions, individually estimated during 9 days from D5 to D13, ranged from 1.33 to 2.62 kg organic matter (OM)/100 kg BW and 1.42 to 2.79 kg DM/100 kg BW (Fig. 3a). Average daily ingestions measured from D5 to D9 (1.99 kg OM/100 kg BW and 2.11 kg DM/100 kg BW) were significantly higher than those measured from D11 to D13 (1.61 kg OM/100 kg BW and 1.72 kg DM/100 kg BW; *P* < 0.0001). On average, animals ingested 1.84 ± 0.06 kg OM/100 kg BW and 1.95 ± 0.07 kg grass DM/100 kg BW (mean ± s.e.m.). The total faecal output was on average 1.51 ± 0.03 kg OM/100 kg BW and 1.76 ± 0.07 kg DM/100 kg BW (mean ± s.e.m.). Expressed in OM, it was significantly higher on D7, D9, D13 than on D11 (1.57 vs 1.39; *P* < 0.01); expressed in DM, there was a tendency to observe the same differences (1.87 vs 1.49; *P* = 0.078). OM digestibility of ingested grass was on average 68.9% but significantly decreased across time. It was significantly higher on D5–D9 (71.1%) than on D11 (67.8%) and D13 (63.7%); the difference between D11 and D13 being also significant (*P* < 0.0001; 94% OM in the grass). The evolution of grass ingestions across time was related to the progressive decrease in both the amount of grass available and its digestibility. The observation at the end of the experiment that the remaining grass was only a ‘carpet’ of stems lying on the ground suggests that animals consumed more and more of the more fibrous parts of the grass which results into a decrease in OM digestibility.

### Soil ingestion

Ti contents in soil (STi), faeces and washed grass were 3821, 157 and 2.6 µg/g DM, respectively. The daily faecal amount of titanium (FATi) and the daily titanium intake due to grass intake (TiIG) (obtained using titanium content in washed grass) were on average 102.0 ± 8.1 and 5.0 ± 0.2 mg Ti/100 kg BW, respectively (mean ± s.e.m.). Daily soil ingestion did not significantly increase along the 9 days (i.e. D5–D13) when the six bulls grazed on the same surface and was on average 26.9 ± 2.6 g DM/100 kg BW (mean ± s.e.m.; *P* = 0.224; Fig. 3b) corresponding to 1.4 ± 0.1% of their total daily DM ingestion. There was a tendency for daily soil ingestion, expressed in percentage of total ingested DM, to increase over time (*P* = 0.112) with daily averages ranging from 1.1 (D5) to 1.4 (D13), the highest value being 1.5 at D11 (Fig. 3c). This tendency can be explained by a lower grass ingestion on the last days of measurements (D11–D13). Nevertheless, there was a high degree of interindividual variability with median, minimal and maximal daily soil ingestions of 27.1, 7.9 and 58.4 g DM/100 kg BW, and 1.3, 0.4 and 2.7% of total ingested DM, respectively (Fig. 3b and 3c). Previous studies conducted in tether-grazing growing bulls already reported a large range of variability in soil ingestions^5,6^ (Table 1). Given that in these experiments the animals presented similar age and BW, this interindividual variability can be attributed to differences in feeding behaviour between individuals.

**Table 1:** Soil ingestions estimated in Creole of Guadeloupe growing bulls at tether-grazing during three experiments (s.e.m.: standard error of mean).

Grazing conditions	Soil ingestion (g DM/100 kg BW/day)					Pregrazing sward height (cm)	References
	Mean	s.e.m	Median	Min	Max		
Long chain	26.9	2.6	27.1	7.9	58.4	49.2	The present study
DRY-ADLIB	55.3	14.4	35.9	21.2	194.1	10.8	Collas et al.^6^
DRY-HIGH	51.2	11.8	37.1	8.2	134.9		
DRY-LOW	61.3	15.6	40.3	13.6	195.4		
HUM-ADLIB	49.5	5.5	48.1	26.8	80.1		
HUM-HIGH	81.3	11.2	89.3	32.0	141.9		
HUM-LOW	82.3	10.9	85.4	30.4	147.4		
NRES	78	13.1	75.7	35.9	133.0	8.4	Jurjanz et al.^5^
RES	98	17.2	88.9	50.2	172.9		

In the study of Collas et al.^6^, the grazing conditions corresponded to three daily herbage allowances (100, 150, 300 g DM/kg BW^0.75^, respectively called LOW, HIGH, ADLIB) performed with water-saturated (HUM) or dry (DRY) soil surface. In the study of Jurjanz et al.^5^, the grazing conditions corresponded to two daily herbage allowances (71 and 128 g DM/kg BW^0.75^, respectively called RES and NRES).

The pregrazing sward height was substantially higher than these two previous studies (Table 1), which is consistent with the idea suggested by Jurjanz et al.^5^ and Collas et al.^6^ that the higher the grass, the lower the soil ingestion. In dairy cows strip grazing in spring on temperate pastures, the highest soil ingestion (0.63 vs 0.17 kg DM/day) had been associated to the lowest post-grazing sward height (3.6 vs 5.3 cm) and to the lowest daily pasture allowance (20 vs 35 kg DM/cow above ground level^8^). In another trial with strip grazing in autumn and different daily pasture allowances (40 vs 65 kg DM/cow above ground level), these authors reported soil ingestions of 0.85 and 0.64 kg DM/day for post-grazing sward heights of 4.1 and 4.7 cm, respectively. Kirby and Stuth^9^ showed soil ingestions from 0.28 to 0.84 kg DM/day by growing steers in May and June in the arid Central Texas. In Idaho, soil ingestions of 0.73 and 0.99 kg DM/day were estimated by Mayland et al.^10^ for cattle at grazing in June and August, respectively. While studies in cattle generally reported soil ingestions up to 10% of the daily ingested DM, studies in sheep can show higher rates. For example, studying soil ingestion by sheep from January to July, Abrahams and Steigmajer^11^ reported in March a median soil ingestion of 17.6% of the daily ingested DM (with individual values from 4.7 to 45.1%). These authors observed the lowest rates in July with a median soil ingestion of 1.5%. These studies suggest that not only the animal species, but also several confusing factors as season and weather conditions, type of soil, and grazing practices may influence soil ingestion by ruminants.

While literature reported soil ingestion by cattle reaching 10% of the daily ingested DM^5,8^, the consequences on animal health, or on the diet digestibility, of such soil ingestions remain poorly documented to date. In chickens, high soil ingestion has been shown to decrease diet digestibility^12^. Others have mentioned the role of soil ingestion in pathogen exposure^13^, and its interest for a mineral supply or to buffer the pH of the digestive system^14,15^, particularly in wildlife. However, soil ingested by herbivores would contribute to their exposure to environmental pollutants (especially organic pollutants as chlordecone that have a high affinity with soil OM^4^) and to the transfer to animal tissues. Contamination of food products of animal origin would limit the sustainability of economic activities related to livestock for farms located in contaminated areas.

### Grass soiling

**Figure 4:**
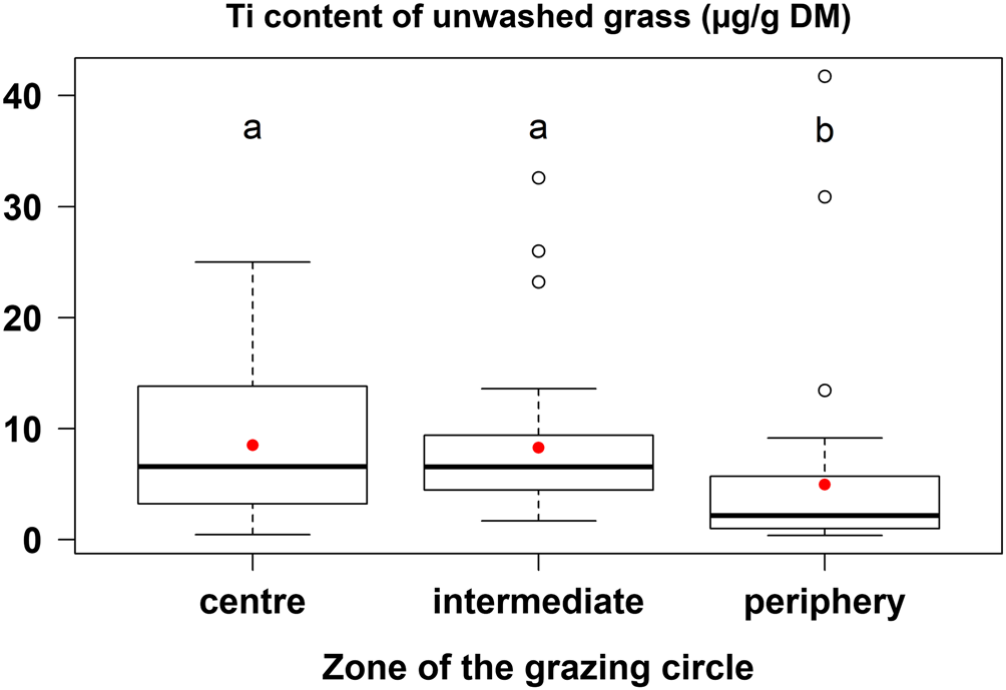
Titanium (Ti) contents of unwashed grass sampled every two days from D2 to D12 in the three zones of each grazing circle (central, intermediate and peripheral areas, respectively 0–3.4 m, 3.4–6.8 m and 6.8–8 m from the stake; n = 36; N = 108) (red points correspond to the means; a, b correspond to differences at *P* < 0.05) (*DM* dry matter).

Regardless of sampling location in the grazing circle, titanium content of unwashed grass did not present temporal variation throughout the experiment with an average of 7.2 µg Ti/g DM (*P* > 0.05). Contrarily, the titanium content of unwashed grass differed significantly between the three different sampling zones in each circle: samples from the peripheral area (5.0 µg Ti/g DM) were significantly lower than those in the intermediate (8.8 µg Ti/g DM; *P* = 0.007) and central zones (8.5 µg Ti/g DM; *P* = 0.013; Fig. 4). There were no significant differences between the titanium contents of grass sampled in the central and in the intermediate zone. The lesser extent of sward soiling in the peripheral area of the grazing circle suggests that animals would have explored the grass resource from the centre to the border of the circle. Thus, the peripheral area would be less soiled due to less trampling. In addition, the chain may not be in contact with the ground in the peripheral area depending on the head movements of the animal. Hinton et al.^16^ observed an increase in soil loading on grass with increasing intensity of grazing by sheep by comparing several stocking rates. The results of the present study suggest that the animals would have ingested the most appetent and not very soiled grass first and, in this way, would have limited their soil ingestion throughout their stay on the same grazing circle.

The differential of titanium contents between washed and unwashed grass evidences that soil particles may adhere to leaves and stems, so that soiled grass ingestion also contributes to soil ingestion. Titanium contents of unwashed grass ranged from 5.0 (periphery of the grazing circle) to 8.8 (intermediate zone of the grazing circle) µg Ti/g DM. Subtracting the average titanium content of the washed grass (2.6 µg Ti/g DM) gives information on the sward soiling induced by grazing with values from 2.4 to 6.2 µg Ti/g DM depending on the zone of the grazing circle. Considering a daily grass ingestion of 1.95 kg DM/100 kg BW and an average titanium content of 3821 µg Ti/g dry soil, the sward soiling estimated during this experiment could induce a daily titanium ingestion from 4.7 to 12.1 mg Ti, and a daily soil ingestion from 1.23 to 3.17 g DM/100 kg BW depending on which zone of the circle is grazed. In this experiment, indirect soil ingestion due to soiled grass represented 4.6–11.8% of the total soil ingestion (considering an average daily soil ingestion of 26.9 g DM/100 kg BW). The representativeness of the unwashed grass samples analysed for their titanium contents compared to the samples of ingested grass can be discussed and the previous estimation of indirect soil ingestion did not allow to quantify precisely the contribution of soiled sward to the total soil intake. Nevertheless, the study of sward soiling gives elements for a better understanding about where the ingested soil comes from. It suggests that indirect soil ingestion, via soiled grass, exists and would contribute to the total soil ingestion, and by consequence, to the exposure to pollutants if the grazing area would be contaminated.

Soil particles may adhere on grass with transport via suspension, or soil disturbance by animal trampling or rainfall splashes^17^, and be retained for several days (above 53 µm) to over a week (below 53 µm) according to the size of soil particles^18^. Sheppard^19^ reported that soil particles which resiliently adhere to plant leaves are generally clay-sized (i.e. < 2 μm). Several authors also used titanium to estimate soil load on grass^20^, and Sheppard et al.^21^ mentioned the interest of rare earth elements. The presence of elements not readily absorbed on plants highlighted the adhesion of soil particles on grass. The titanium content of washed grass was due to soil particles which resisted despite grass washing.

The sward soiling increases when animals grazed on water-saturated surface compared to dry soil surface, as shown by Collas et al.^6^ and measured also by the titanium content of unwashed grass. In the present study, daily rainfall was recorded and was not significantly linked to titanium content in unwashed grass (*P* > 0.05). The recorded daily rainfall showed higher values for the four last days (D9–D12) on the experimental plot whereas sward soiling did not change during the whole experiment. This study did not allow to evidence a link between daily rainfall and sward soiling but soil surface moisture was neither controlled nor measured, contrary to the study of Collas et al.^6^ for which the two levels of soil surface moisture were well contrasted because of an experimental irrigation of 20 mm per night.

## Conclusion

The present study completed the approach of soil ingestion in tethered growing cattle with estimation of soil ingestion when tethered cattle stay during 11 days on the same plot with a large surface (‘long chain’) and a very high sward as it is frequently met in grazing practices among Caribbean breeders. The results confirmed the importance of sward height suggested by previous studies, and demonstrated that soil ingestion can be reduced when animals graze on high swards. Thus, sward height is an effective indicator to limit animal exposure in contaminated areas in order to preserve livestock farming activities and their supply service in healthy animal products. Nevertheless, it is necessary to find a trade-off between sward height and nutritional quality of grass. This study was also the first, which deepened the effect of sward soiling dynamics over 11 days and evidenced a differential soiling according to the distance from the stake. In the peripheral area, the sward was significantly less soiled by soil particles adhered on leaves or stems than in the central and intermediate areas. This can be explained by trampling all the more important as the animal is close to its stake. The action of grazing and trampling laid the long stems on the ground forming an insulating mat between ground and animal. This must have limited the direct soil ingestion by the cattle. In contrast, analyses on unwashed grass implied indirect exposure through ingestion of soiled plants. Several studies were conducted in experimental controlled conditions and allowed to understand variation factors of soil ingestion by cattle although there is a strong inter-individual variability. It would be interesting now to compare these estimations with a range of soil ingestions individually estimated in ruminants in non-experimental tropical conditions.

## Methods

The experiment was performed at the Tropical Platform for Experimentation on Animals of INRAE (Centre Antilles-Guyane, Petit-Bourg, Guadeloupe, French West Indies; 16° 12′ N, 61° 40′ W; altitude of 111 m).

The whole experiment was approved in advance by the Ethics Committee for Animal Experimentation of The French West Indies and French Guiana (C2EA-69) and the French Ministry of National Education, Higher Education and Research, and registered under number APAFIS#552720160508133139. The whole experiment was performed in farm-like conditions and in accordance with relevant guidelines and regulations.

### Treatments and experimental design

Six 18–20 months old bulls (270 ± 14 kg BW; mean ± s.e.m) of the Creole of Guadeloupe breed (*Bos taurus*) were tethered with a 8-m chain in a Lucuntu grass (*Ischaemum timorense Kunth*) based pasture whose regrowth had reached near to 50 cm (i.e. average pregrazing sward height of 49 cm). Thus, the animals could graze on a 200-m^2^ circular surface (called ‘grazing circle’). Water was available ad libitum on each tethering placement.

The experiment lasted 13 days (14th to 26th November 2017) with 4 days of adaptation (i.e. D1–D4) to experimental conditions and 9 days of ingestion measurements and sampling (i.e. D5–D13). Animals had been previously adapted to tether grazing. The morning of D2, animals were moved on the experimental plot until D12. The morning of D13, they were moved on a new plot the time to collect the last daily faecal samples on the experimental plot. Taking into account the digestive transit time in ruminants^22,23^, the faecal samples collected every morning from D5 to D13 on the experimental plot corresponded to what the animals mainly ingested from D2 to D10 on the experimental plot.

During the period from D2 to D12, mean daily rainfall was 2.8 ± 0.9 mm, with most precipitation over the last 4 days (D2–D8: 0.9 ± 0.5 mm; D9–D12: 6.3 ± 0.6 mm). Daily Penman evapotranspiration^24^ was 3.8 ± 0.2 mm, and minimum and maximum temperatures were on average 21.8 ± 0.3 and 29.8 ± 0.2 °C, respectively (mean ± s.e.m.).

### Measurements

Sward height of each grazing circle was determined daily on the experimental plot (i.e. D2–D12) using a rising plate herbometer (HERBOMETRE designed and developed by ARVALIS—Institut du végétal). The herbometer was modified to be lengthened to measure very high swards (up to 63 cm at D2). Seven individual sward height measurements, spaced by 1.1–1.2 m from each other, were realised along a line from the centre to the edge of the circle. This was repeated on three lines spaced by a 120° angle to obtain 21 sward height points daily for each grazing circle.

### Sampling, sample preparation and analyses

At the beginning of the experiment, a grass sample representative of the offered sward was taken in the experimental plot, out of the grazing circles, then washed in warm water to evacuate soil traces before drying. Soiling of the sward was evaluated from grass sampled every two days from D2 to D12 at three zones (central, intermediate and peripheral areas) in the circle of each animal. The central, intermediate and peripheral areas were separated by two virtual circles at 3.4 and 6.8 m from the stake. These grass samples were not washed before drying. Total faecal output, necessary to estimate ingestions of soil and grass, was individually assessed by collecting and weighing all dung pats for each animal daily from D5 to D13. A 200 g sample was daily constituted for each animal taking fresh matter from the core of the dung pat to avoid soil or dust contamination. Individual samples were collected before the total dung collection from each animal was completed. Indeed, faeces contamination with soil or dust would result in a higher faecal titanium content (used as a soil marker, see “Calculations” section), and therefore overestimate soil ingestion. All faecal samples were used to estimate the DM content in order to obtain total faecal output every day for each animal, whereas chemical analyses were realised only on faecal samples collected every other day (i.e. D5, D7, D9, D11, D13). All grass and faeces samples were dried at 60 °C until constant weight (approximately 48 h for grass and 72 h for faeces samples). The grass and concerned faecal samples were milled (< 1 mm) and analysed for ash (incineration at 550 °C during 6 h) and titanium (Ti) contents. Faecal samples were also analysed for crude protein contents (Dumas method^25^). Surface soil (0–5 cm depth) was randomly sampled in each circle at the end of the trial, and then wet sieved at 2 mm to remove roots and coarse elements, dried (60 °C until constant weight, approximately 96–120 h) and milled (< 100 µm) before Ti analysis. Ti was analysed in grass, faeces and soil by ICP-MS method according to Collas et al.^6^ (inductively coupled plasma mass spectrometry).

### Calculations

Individual daily grass DM ingestions were estimated as described previously by Jurjanz et al.^5^ calculating first individual daily OM ingestions from faecal output over 24 h and then OM digestibility from faecal crude protein content^26^. Individual daily soil ingestions were expressed in g DM per 100 kg BW and calculated with titanium as a soil marker using the following equation:

$${\text{Individual daily soil ingestion}}=({\text{FATi}} - {\text{TiIG}}) / {\text{STi}},$$

where FATi is the daily faecal amount of titanium, obtained with faecal titanium content and daily faecal output expressed per 100 kg BW; TiIG is the daily titanium ingestion due to grass ingestion, obtained with titanium content in the washed grass sample (to not consider titanium ingestion due to ingestion of soil particles adhered on grass) and daily DM ingestion expressed per 100 kg BW; and STi is the soil titanium content. Individual daily total DM ingestions was then estimated by adding individual daily soil ingestions from individual daily grass DM ingestions. Individual daily soil ingestions were then expressed in percentage of daily total DM ingestion.

### Statistical analyses

Statistical analyses were performed using R software (version 3.6.1^27^). Sward height measurements were tested using a linear mixed model with date (i.e. days of measurements from D2 to D12) as a fixed effect, and animals repeated on date as a random effect. The eleven dates were compared by the means using Tukey correction (*P* < 0.05). All variables related to soil and grass (dry and organic matter) ingestion measurements were tested using linear mixed models with date (i.e. days of measurements from D5 to D13) as a fixed effect, and animals repeated on date as a random effect. Animals were used as the experimental unit. As soil ingestion was estimated every two days, the smooth.spline function of R was used to smooth the individual temporal evolution curves in the graphical representations. Ti contents of unwashed grass (N = 108), used as an indicator of grass soiling (estimated every two days from D2 to D12), were tested using a linear mixed model with zone in the grazing circle (central, intermediate or peripheral area; n = 36 values per zone), sampling date (D2, D4, D6, D8, D10, D12), zone × sampling date interaction, and mean daily rainfall as fixed effects, and animals repeated on date as a random effect. The effects of mean daily rainfall, sampling date and zone × sampling date interaction were removed from the final model for grass soiling as they were not significant. The three levels of the zone factor were compared by the means using Tukey correction (*P* < 0.05).
